# Efficacy and safety of PARP inhibitors in advanced or recurrent endometrial cancer: a systematic review and meta-analysis

**DOI:** 10.3389/fimmu.2025.1659650

**Published:** 2026-01-07

**Authors:** Huanhuan Zhang, Guangwei Yan, Jinxiang Yan, Xianxu Zeng

**Affiliations:** 1 Department of Pathology, The Third Affiliated Hospital of Zhengzhou University, Zhengzhou, China; 2 Zhengzhou Key Laboratory of Gynecological Disease’s Early Diagnosis, Zhengzhou, China

**Keywords:** endometrial cancer, immunotherapy, meta-analysis, PARP inhibitors, PD-L1 inhibitor

## Abstract

**Objective:**

Several clinical trials have explored the efficacy and safety of Poly (ADP-ribose) polymerase (PARP) inhibitors in endometrial cancer (EC). However, evidence supporting PARP inhibitors alone or in combination with other medications in advanced or recurrent EC remains limited.

**Methods:**

We utilized Cochrane Library, PubMed, Web of Science, and Embase to identify clinical trials that evaluated the efficacy and safety of PARP inhibitors in advanced and recurrent EC. The outcomes analyzed included progression-free survival (PFS), overall survival (OS), objective response rate (ORR), and adverse events (AEs). We computed hazard ratios (HRs) for PFS and OS, with 95% confidence intervals (CIs) from randomized trials. Subgroup analysis was conducted based on the PARP inhibitor combination therapy strategies (with antiprogrammed death 1 [PD-1]/antiprogrammed death ligand-1 [PD-L1] inhibitors or antiangiogenic agents).

**Results:**

Overall, 12 clinical trials for a total number of 1,594 patients diagnosed with advanced or recurrent EC were included in this meta-analysis. The duration of follow-up time ranged from a median of 7.4 to 31.9 months, and the pooled median PFS was 6.43 months. The results showed that the combination of PARP with PD-L1/PD-1 inhibitors could significantly prolong PFS versus placebo (hazard ratio [HR] = 0.567; 95% confidence interval [CI] = 0.469–0.686; *p* < 0.05). Based on the subgroup analysis, the pooled median PFS was found to be 7.59 months for the combination of PARP with PD-L1/PD-1 inhibitors. In addition, the pooled median PFS at 6 months was 0.44 based on the analysis of four included studies. In the intention-to-treat population, there was no PFS/OS difference between PARP inhibitor monotherapy and placebo (PFS: HR = 0.684 [95% CI = 0.334–1.398], *p* > 0.05; OS: HR = 0.787 [95% CI = 0.279–2.219], *p* > 0.05). The ORR was notably elevated in the combined therapy group compared with PARP inhibitor monotherapy (OR = 3.34 [95% CI = 1.095–10.165], *p* = 0.034). The most common AEs included fatigue (226, 54.5%), nausea (219, 52.8%), anemia (219, 52.8%), diarrhea (127, 30.6%), and alopecia (121, 29.2%).

**Conclusion:**

The combination of a PARP inhibitor with a PD-L1/PD-1 inhibitor shows modest activity with substantial toxicity, particularly in heavily pretreated and largely pMMR populations.

**Systematic review registration:**

https://www.crd.york.ac.uk/PROSPERO/, identifier CRD42024556356.

## Introduction

Endometrial cancer (EC) is the most common gynecological malignancy in developed countries, with a majority of cases occurring postmenopause (1). Over the past decade, the prevalence of EC and disease-associated mortality rates has been continuously rising worldwide (1, 2). The prognosis of patients with primary advanced EC is poor, and available therapeutic options are limited. At the moment, no standard therapy has been globally accepted as the standard of care for advanced and recurrent EC after the failure of platinum-based chemotherapy.

Since the proposal of molecular subgroups based on the analysis of the Cancer Genome Atlas (TCGA) in 2013, it has played a crucial role in formulating the most appropriate therapy and identifying prognostic factors (3). More recently, monotherapy with an immune checkpoint inhibitor (ICIs), including antiprogrammed death 1 (PD-1) and antiprogrammed death ligand-1 (PD-L1), has demonstrated a substantial improvement in oncologic outcomes in patients with deficient mismatch repair (MMRd) EC (4, 5). Although several trials highlighted the limited efficacy of anti-PD-1/PD-L1 agents alone, combined therapy of PD-1/PD-L1 inhibitors and antiangiogenic agents is an attractive approach in proficient mismatch repair (pMMR) EC (6, 7).

In 2009, a phase I study presented the first clinical evidence of olaparib, a PARP inhibitor, having antitumor activity in patients associated with breast cancer (BRCA)1/2 mutations (8). In patients without BRCA mutations, the therapeutic value of PARP inhibitors is associated with homologous recombination deficiency (HRD) (9). Defects in homologous recombination repair (HRR) genes, including BRCA1/2, BRIP1, and RAD51C, have the potential to produce HRD (10, 11). A systematic review reported that BRCA1/2 pathogenic variants were identified in 4.3% of EC patients (10). In a large-scale study of molecular profiles of 1,475 EC cases, pathogenic variants in HRR genes were observed in 34.4% of cancers (12). Finally, alterations in the phosphatase and tensin homolog (PTEN) gene occur in up to 78% of EC (13). In preclinical studies, loss of PTEN is associated with HRD, sensitizing tumors to PARP inhibitors (14).

Although several clinical trials have either been completed or are ongoing, evidence supporting the PARP inhibitors, alone or in combination with other drugs, in advanced EC still remains limited. Currently, the majority of relevant studies are phase II clinical trials with small sample sizes. As such, this meta-analysis aimed to synthesize the available data to investigate the effectiveness and safety of PARP inhibitors for the treatment of advanced or recurrent EC.

## Materials and methods

### Search strategy and selection criteria

This systematic review was conducted and reported in accordance with the PRISMA 2020 guidelines. The Preferred Reporting Items for Systematic Reviews and Meta-Analyses (PRISMA) 2020 checklist is presented in the **Supplementary materials**. Electronic databases (PubMed, Embase, Web of Science, and Cochrane databases) were searched from inception up to 25 November 2025. Search terms included (“PARP inhibitor” OR “PARPi” OR “poly ADP-ribose polymerase inhibitor” OR “olaparib” OR “rucaparib” OR “veliparib” OR “niraparib” OR “talazoparib”) AND (“endometrial cancer” OR “endometrial carcinoma” OR “endometrium cancer” OR “endometrial neoplasms” OR “endometrial neoplasms”). In addition, references of related articles were searched to identify other relevant publications.

The inclusion criteria for studies in this meta-analysis were as follows: (1) patients diagnosed with advanced or recurrent EC who underwent treatment with a PARP inhibitor either alone or combined with PD-1/PD-L1 inhibitor or antiangiogenic agents; (2) studies with adequate clinical information, including the name of the medication used, the total number of patients, objective response rate (ORR), progression-free survival (PFS), overall survival (OS), and occurrence of adverse events (AEs); (3) study designs including cohort studies, case–control studies, randomized controlled trials (RCTs), or single-arm trials. For studies reporting on the same populations, we included the most recent results. The principal investigator (HH Z) and the second author (GW Y) independently scrutinized eligibility criteria and extracted study characteristics. This meta-analysis was registered in PROSPERO (registration number: CRD42024556356).

### Data extraction and quality assessment

We summarized the characteristics of selected publications, including first author, year of publication, study phase, ClinicalTrials.gov identifier, follow-up time, total number of participants, PFS, OS, ORR, and AEs. For studies involving multiple tumors, only the endometrial cancer subset was included. The quality of the evidence was assessed using the Cochrane risk-of-bias tool (RoB 2) for randomized trials (RCTs) and the risk of bias in nonrandomized studies of interventions (ROBINS-I).

### Statistical analysis

The median times and hazard ratios (HRs) for OS and PFS, with their 95% confidence intervals (95% CI), were extracted from the included clinical trials. Pooled estimates of both efficacy and safety outcomes were calculated when ≥ 2 clinical trials reported the results of the same prespecified outcome. Specifically, pooled estimates were calculated using the inverse variance method (metamean for PFS/OS, metaprop with logit transformation for ORR and AEs; metagen for log HRs for PFS/OS). The combined odds ratio (OR) and 95% CI were converted to an incidence rate to assess the ORR of PARP inhibitors. *p* < 0.05 for the Cochran *Q* estimates or *I^2^* > 50% was considered to indicate high heterogeneity, and the random-effects model was applied; otherwise, the fixed-effects model was used. The effect model and heterogeneity statistics for each analysis are shown in **Table 1**. Publication bias was not assessed due to the small number of clinical trials (< 10) included (15). Sensitivity analysis was performed to assess the influence of the DUO-E trial and RUBY trial on the meta-analysis results. *p* < 0.05 was considered to indicate a statistically significant difference. Statistical analysis was performed using the “meta” package in R software.

**Table 1:** Meta-analysis of the efficacy of PARP inhibitors in the treatment of advanced or recurrent endometrial cancer.

Outcomes and subgroups	Number of studies	Meta-analysis			Heterogeneity			
		Pooled estimates (95% CI)	HR (95% CI)	*p-*value	*I^2^*	Tau^2^	*p*-value	Analysis model
PFS								
Overall	9	6.434 (3.536–9.332)	–	–	#####	18.2	< 0.0001	Random-effects
PARP with PD-L1/PD-1 inhibitors	5	7.585 (2.059-13.110)	–	–	#####	38.062	< 0.0001	Random-effects
PARP inhibitors vs. placebo	2	–	0.684 (0.334–1.398)	0.297	#####	0.2	0.0512	Random-effects
PARP with PD-L1/PD-1 inhibitors vs. placebo	2	–	0.567(0.469-0.686)	< 0.0001	0	0	0.669	Fixed-effects
**PFS6 (overall)**	4	0.440(0.143-0.737)	–	–	#####	0.087	< 0.0001	Random-effects
OS								
Overall	4	11.837 (3.124–20.549)	–	–	#####	73.45	< 0.0001	Random-effects
PARP inhibitors vs. placebo	2	–	0.787 (0.279–2.219)	0.651	#####	0.443	0.034	Random-effects
ORR								
Overall	8	0.171 (0.123–0.219)	–	–	#####	< 0.0001	0.349	Fixed-effects
PARP with PD-L1/PD-1 inhibitors	3	0.138 (0.072–0.203).	–	–	0.00%	0.00%	0.829	Fixed-effects
PARP with antiangiogenesis	2	0.227 (0.064–0.389)	–	–	#####	0.008	0.11	Random-effects
PARP inhibitors combined therapy vs. monotherapy	2	–	0.300 (0.098–0.913)	0.034	0%	0	0.903	Fixed-effects
AEs								
Any grade	2	0.947 (0.835–1.00)	–	–	#####	0.006	0.121	Random-effects
AEs of grade ≥ 3	2	0.720 (0.603–0.838)	–	–	0.66	0.005	0.086	Random-effects

*PFS*, progression-free survival; *OS*, overall survival; *PD-L1*, programmed cell death receptor ligand 1; *HR*, hazard ratio; *CI*, confidence interval; *PFS6*, PFS at 6 months.

## Results

### Study selection and characteristics

**Figure 1:**
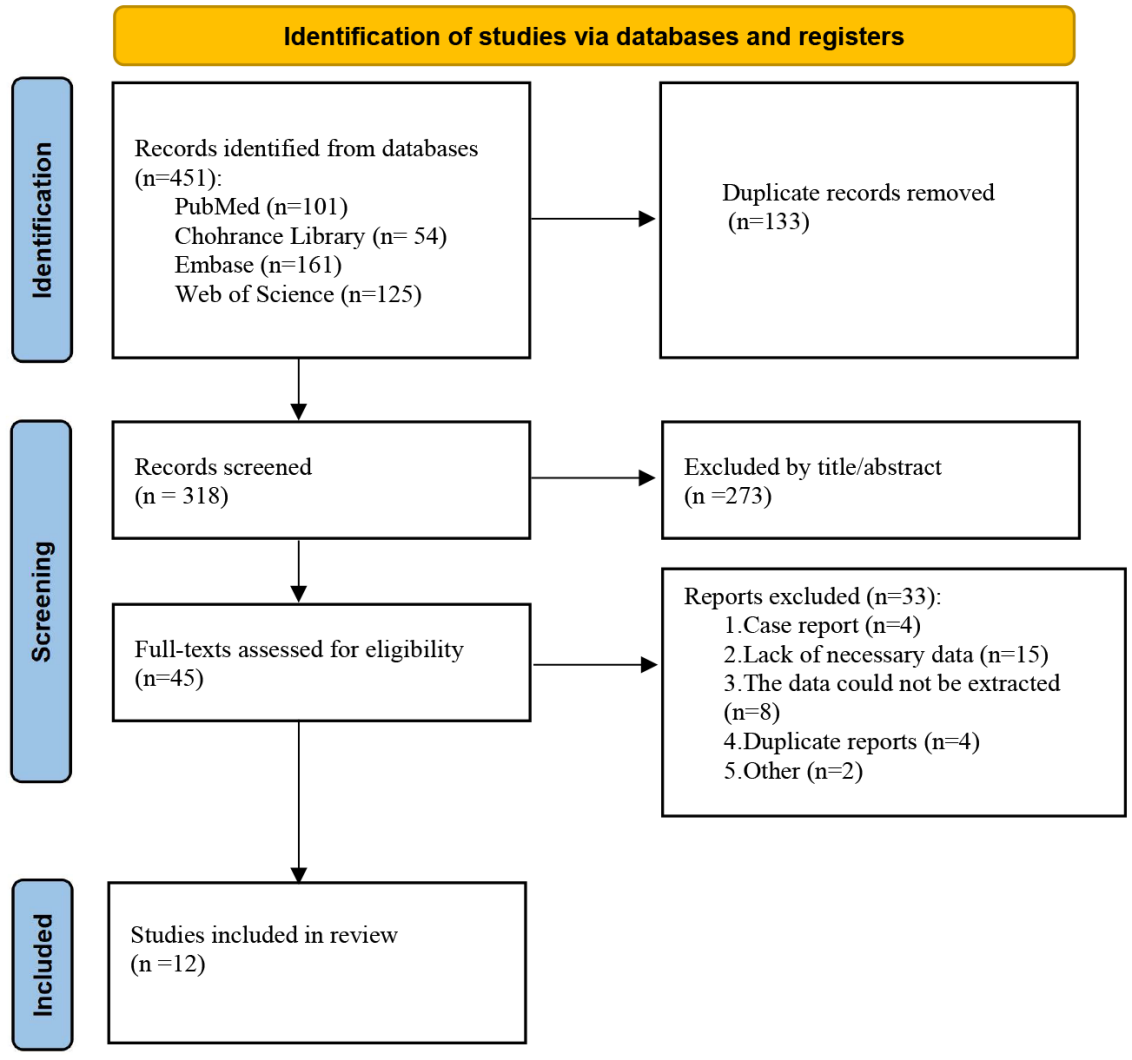
PRISMA flow diagram illustrating the literature selection process.

The literature search process is shown in **Figure 1**. Based on the inclusion criteria, 12 clinical trials including a total of 1,594 patients diagnosed with advanced or recurrent EC were included in this meta-analysis (**Table 2**). (16–26). All studies were published between 2021 and 2025. These studies included one clinical phase Ib trial, nine phase II trials, and two phase III trials. Specifically, five trials investigated the combination of PARP with PD-L1/PD-1 inhibitors, and two trials investigated the combination with an antiangiogenesis agent. Two trials examined both PARP inhibitor monotherapy and combination therapy (17, 21). Data from the combination therapy were used for the analysis.

**Table 2:** Baseline characteristics of patients in the trials included in the meta-analysis.

	Author (year)	Clinical trial	Study design	Sample size	Population	Experimental arm	Control arm (*n*)	Endpoints	ORR	HR (95% CI)-PFS	Median PFS (months, median; 95% CI)	6-month PFS rate (%; 95% CI)	HR (95% CI)-OS	Median OS (months, median; 95% CI)	Follow-up time (median/months)
1	Jackson (2022) (16)	NCT03476798	Phase II	27	Recurrent EC	Bevacizumab + rucaparib	–	ORR, PFS6	14% (4/27)	–	–	30 (10–50)	–	–	25 months
2	Madariaga (2022) (17)	NCT03016338	Phase II	57	Recurrent EEC/SE	Niraparib + dostarlimab (22)	Niraparib (25)	ORR, DCR, PFS, OS	4% (1/25)	–	2.5 (1.8–3.7)	–	–	12.5 (6.6–19.3)	–
3	Westin (2024) (18)	NCT04269200 (DUO-E Trial)	Phase III	718	Advanced/metastatic EC	Durvalumab + olaparib (*n* = 239), durvalumab (*n* = 238)	Placebo (*n* = 241)	PFS, OS	–	Durvalumab + olaparib: 0.55 (0.43–0.69); durvalumab: 0.71 (0.57–0.89)	Durvalumab + olaparib: 15.1 (12.6–20.7); durvalumab: 10.2 (9.7–14.7); placebo: 9.6 (9.0–9.9)	Durvalumab + olaparib: 87.2 (73.8–94.1); durvalumab: 83.8 (78.4–88.0); placebo: 82.5 (76.9–86.8)	Durvalumab + olaparib: 0.59 (0.42–0.83); durvalumab: 0.77 (0.56–1.07)	Durvalumab + olaparib: NR (NR–NR); durvalumab: NR (NR–NR); placebo: 25.9 (23.9–NR)	Durvalumab + olaparib, durvalumab: 0.71 (0.57–0.89): 15.4 months; placebo: 12.6 months
4	Post (2022) (19)	NCT03951415	Phase II	50	Advanced EC	Durvalumab + olaparib	–	PFS6, ORR, OS	16% (8/50)	–	3.4 (2.8–6.2)	34.0 (23.1–50.0)		8.0 (7.5–14.3)	17.6 months
5	Konstantinopoulos (2022) (20)	NCT02912572	Phase II	35	Recurrent pMMR EC	Talazoparib + avelumab	–	PFS6, ORR	11.4% (4/35)	–	3.6 (2.4–5.4)	22.9 (10.4–40.1)		–	12.9 months
6	Rimel (2022) (21)	NCT03660826	Phase II	120	Advanced/metastatic EC	Olaparib (*n* = 40), olaparib + cediran (*n* = 40)	Cediranib (*n* = 40)	PFS, ORR, OS	Cediranib: 24.1% (7/29); olaparib: 12.5% (4/32); olaparib + cediran:31.4% (11/35)	Cediranib: 1.45 (one‐sided 95% CI, 2.14); olaparib + cediran: 0.7 (95% CI, 1.05)	Cediranib: 3.8 (3–5.4); olaparib: 2.0 (1.8–4.7); olaparib + cediran: 5.5 (3.7–8.3)	–	Cediranib: 1.01 (one‐sided 95% CI, 1.62); olaparib + cediran: 5.5 (3.7–8.3):0.75 (95% CI, 1.25)	Cediranib: 12.4 (7.5–22.9); olaparib: 13.4 (6.5–19.5); olaparib + cediran: 17.6 (9.7–NR)	18.3 months
7	Westin (2021) (22)	NCT02208375	Phase Ib	11	Recurrent EC	Olaparib + capivasertib	–	ORR	44% (4/9)	–	–	–	–	–	7.4 months
8	Poveda (2022) (23)	NCT02684318	Phase II	26	Advanced/metastatic EEC	Lurbinectedin + olaparib	–	ORR, PFS, OS	15.4% (4/26)	–	4.8 (1.9–6.8)	–	–	15.9 (7.2–23.3)	–
9	Bradley (2025) (24)	NCT03617679	Phase II	79	Advanced/metastatic EC	Rucaparib (39)	Placebo (40)	PFS, OS	–	0.45 (0.24–0.87)	Rucaparib: 28.1 (12.8–NR); placebo: 8.7 (5.4–16.7)	–	0.43 (0.18–1.05)	Rucaparib: NR (34.8–NR); placebo: 28.4 (19.0–NR)	25 months
10	Florence (2025) (25)	NCT03745950 (UTOLA trial)	Phase IIb	145	Advanced/metastatic EC	Olaparib (96)	Placebo (49)	PFS, OS	–	0.94 (0.65–1.35)	5.6 (3.7–8.8)	–	1.25 (0.80–1.94)	22.2 (16.0–25.3)	31.9 months
								–	–	–	4.0 (3.5–7.7)	–	–	23.8 (19.6–28.6)	
11	Piffoux (2025) (26)	NCT02755844 (ENDOLA)	Phase I/II	35	Advanced/metastatic EC	Olaparib + metformin	–	PFS, ORR	20.80%	–	5.2 (5.1–8.8)	–	–	–	5.1 months
12	Mirza (2025) (27)	NCT03981796 (RUBY)	Phase III	291	Advanced/metastatic EC	Dostar/CP + dostar/nira (192)	PBO/CP + PBO (99)	PFS	–	0.60 (0.43–0.82)	Dostar/CP + dostar/nira: 14.5 (11.8–17.4); PBO/CP + PBO: 8.3 (7.6–9.8)	–	–	–	19 months

Abbreviations: *NR*, not reached; *PBO/CP + PBO*, placebo + carboplatin–paclitaxel followed by placebo; *Dostar/CP + dostar/nira*, dostarlimab + carboplatin–paclitaxel followed by dostarlimab + niraparib.

The methodological assessment of the included studies is presented in **Supplementary Table S1**. Overall, a low to moderate risk of bias was observed in most included articles. Only one study was found to present a high risk of bias in the selection of participants (**Supplementary Table S1**; **Supporting information**).

### Progression-free survival and overall survival

**Figure 2:**
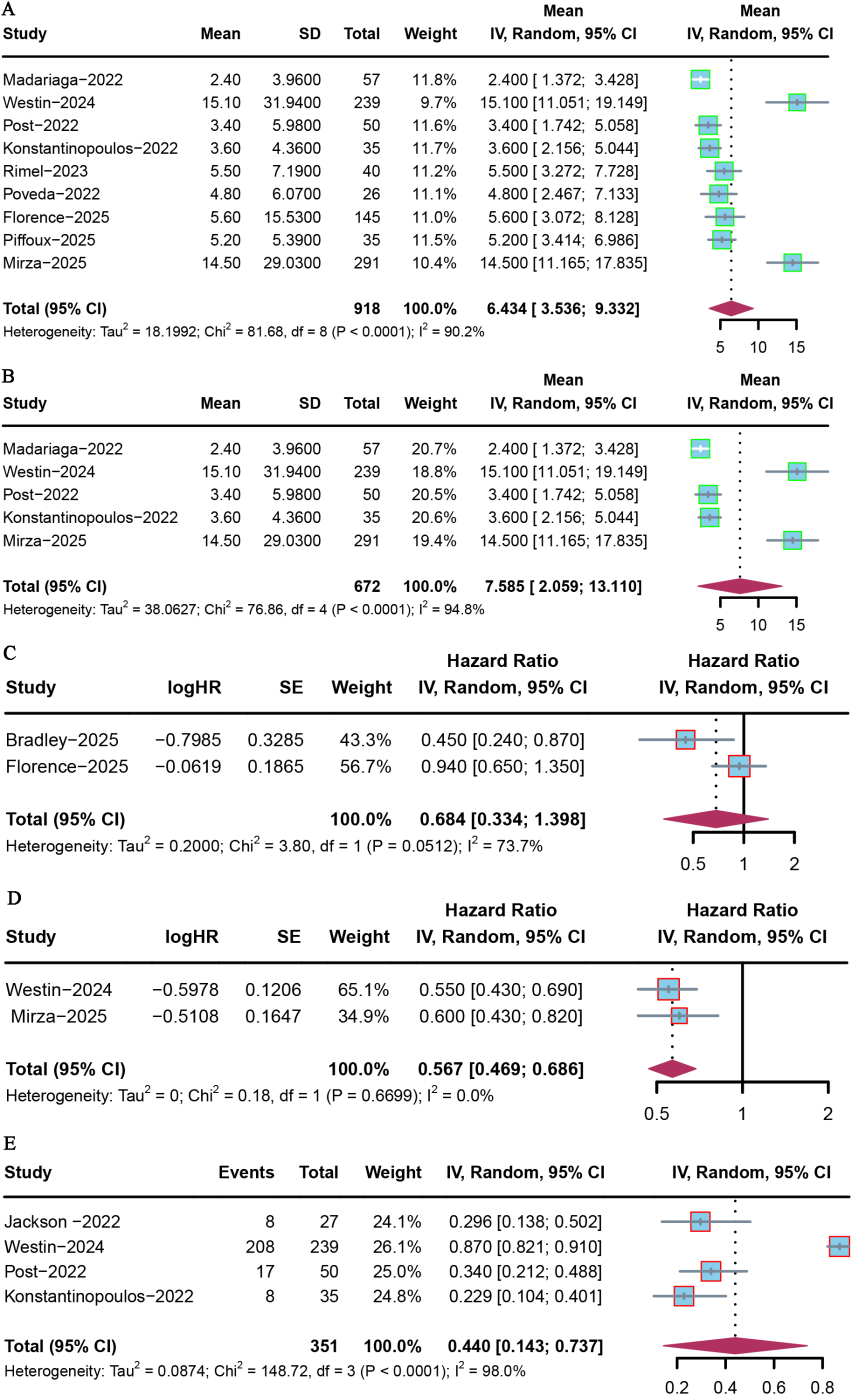
Forest plot of PFS for PARP inhibitor combination therapy in advanced or recurrent EC. **(A)** Overall population; **(B)** PARP inhibitors combined with PD-1/PD-L1 inhibitors; **(C)** PARP inhibitor monotherapy *vs*. placebo; **(D)** PARP combined with PD-L1/PD-1 inhibitors *vs*. placebo; **(E)** PFS6.

The duration of follow-up time ranged from a median of 7.4 to 31.9 months. PFS was reported in nine included studies. Among these, Madariaga et al. reported the lowest PFS of 2.4 months (95% CI = 1.6–3.7) (17), while Westin et al. reported the highest PFS of 15.1 months (95% CI = 12.6–20.7) (18). The pooled median PFS was 6.43 months (95% CI = 3.54–9.33) (**Figure 2A**). In the intention-to-treat population, no PFS difference was observed between PARP inhibitor monotherapy and placebo (HR = 0.684 [95% CI = 0.334–1.398], *p* > 0.05; **Figure 2C**). However, the combination of PARP with PD-L1/PD-1 inhibitors significantly prolonged PFS versus placebo (HR = 0.567 [95% CI = 0.469–0.686], *p* < 0.05; **Figure 2D**).

Based on the subgroup analysis, the pooled median PFS was 7.59 months (95% CI = 2.06–13.11) for advanced or recurrent EC patients who received combined therapy with a PD-1/PD-L1 inhibitor (**Figure 2B**). In addition, the pooled median PFS at 6 months was 0.44 (95% CI = 0.143–0.737), based on the analysis of four included studies (**Figure 2E**).

To further elucidate the high heterogeneity, a sensitivity analysis was performed excluding the phase III DUO-E trial and RUBY trial (18). Exclusion of the DUO-E trial significantly decreased the heterogeneity, and the pooled median PFS was 4.10 (95% CI = 3.09–5.10) and 2.972 (95% CI = 2.121–3.824) for the overall population and for patients who received the combined therapy with a PD-1/PD-L1 inhibitor, respectively (**Supplementary Figure S1**). Although the number of studies was limited, the studies in the funnel plot were symmetrically distributed with respect to the pooled estimate, suggesting no publication bias (**Supplementary Figure S2**).

**Figure 3:**
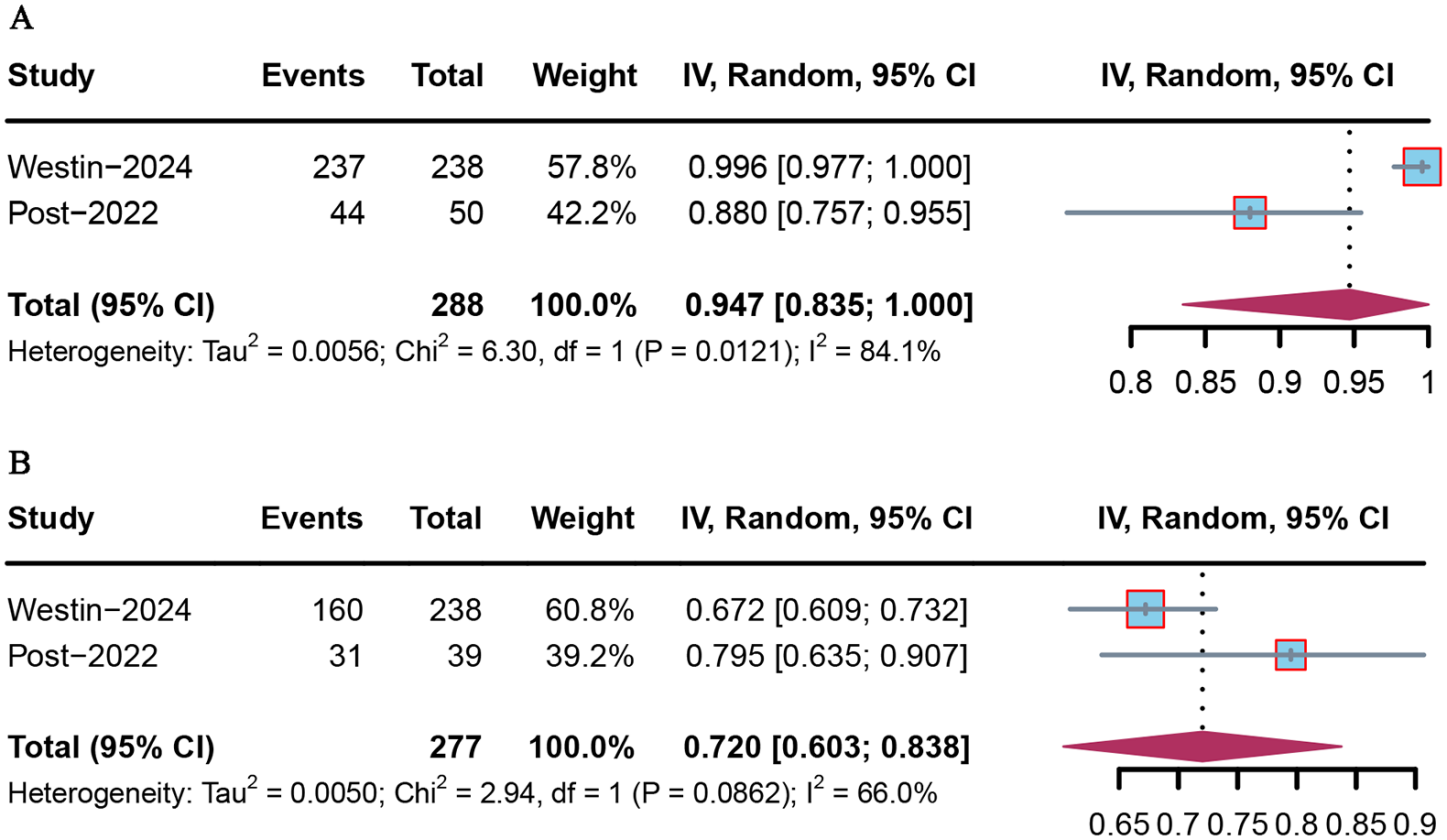
Forest plot of OS for PARP inhibitor combination therapy in advanced or recurrent EC. **(A)** Overall population; **(B)** PARP inhibitor monotherapy *vs*. placebo.

OS data were reported in seven included studies; however, median OS was not reached in three studies. Among the included studies, Post et al. reported the lowest OS at 8.0 months (95% CI = 7.5–14.3) (19), while Westin et al. reported the highest OS as not reached (95% CI = not reached–not reached) (18). The pooled median OS was 11.837 months (95% CI = 3.124–20.549) (**Figure 3A**). In the intention-to-treat population, there was no OS difference between olaparib and placebo (HR = 0.787 [95% CI = 0.279–2.219], *p >* 0.05; **Figure 3B**).

### Objective response rate

**Figure 4:**
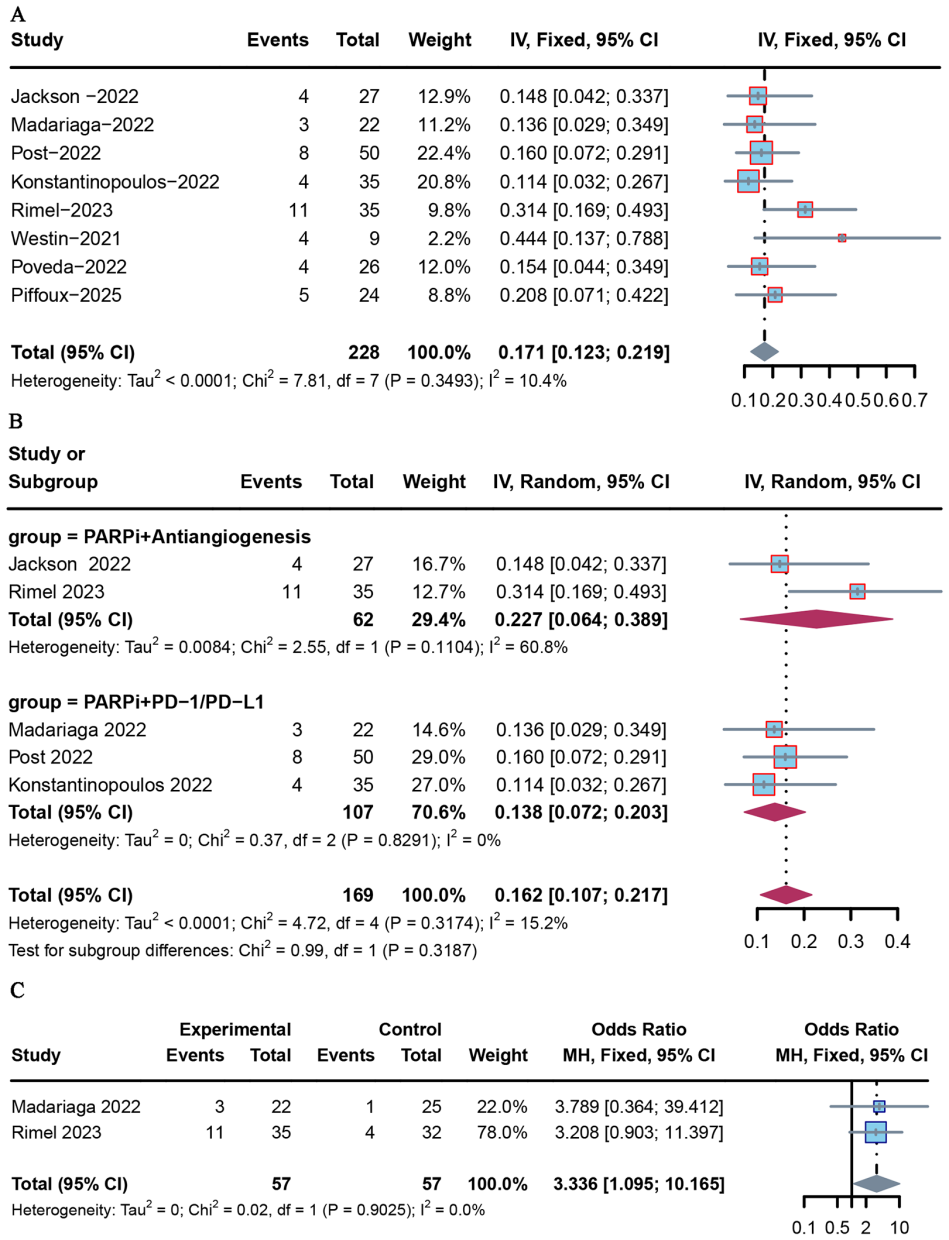
Forest plot of ORR for PARP inhibitor combination therapy in advanced or recurrent EC. **(A)** Overall population; **(B)** patients receiving combination therapy with PD-1/PD-L1 inhibitor and antiangiogenesis agent; **(C)** PARP inhibitor monotherapy versus combination therapy.

ORR data were reported in eight included studies. The pooled ORR was 0.17 (95% CI = 0.123–0.219) (**Figure 4A**). Among these studies, Post et al. reported the lowest ORR (8%) (19), while Westin et al. reported the highest ORR (44.0%) (18). Based on subgroup analysis, the pooled ORRs were 0.138 (95% CI = 0.072–0.203) and 0.227 (95% CI = 0.064–0.389) for advanced or recurrent EC patients who received combined therapy with PD-1/PD-L1 inhibitor and antiangiogenesis agent, respectively (**Figure 4B**). In addition, ORR was notably elevated in the combined therapy group compared with PARP inhibitor monotherapy (OR = 3.34 [95% CI = 1.095–10.165], *p* = 0.034) (**Figure 4C**).

### Adverse effects

Only six included studies provided available AEs data, and the occurrence of AEs of any grade was analyzed in two studies. The overall pooled proportion of AEs was 0.947 (95% CI = 0.835–1.000) (**Supplementary Figure S3A**). AEs of grade ≥ 3 were analyzed in two studies. The overall pooled proportion of AEs of grade ≥ 3 was 0.72 (95% CI = 0.603–0.838) (**Supplementary Figure S3B**).

Details of AEs are summarized in **Table 3**. The most common AEs included fatigue (226, 54.5%), nausea (219, 52.8%), anemia (219, 52.8%), diarrhea (127, 30.6%), and alopecia (121, 29.2%). When considering only AEs of grade ≥ 3, patients had a higher incidence of anemia (88, 21.2%), decreased neutrophil count (76, 18.3%), fatigue (31, 7.5%), decreased platelet count (28, 6.7%), and decreased white blood cell count (17, 4.1%).

**Table 3:** Any-grade and grade ≥ 3 adverse events reported in the included studies in this meta-analysis.

Author/ year	Madariaga (*n* = 22)		Westin (*n* = 238)		Post (*n* = 50)		Konstantinopoulos (*n* = 35)		Rimel (*n* = 39)		Piffoux (31)		Total (*n* = 415)	
	Any grade	Grade 3/4	Any grade	Grade 3/4	Any grade	Grade 3/4	Any grade	Grade 3/4	Any grade	Grade 3/4	Any grade	Grade 3/4	Any grade	Grade 3/4
Any-grade AE	–	–	237 (99.6%)	160 (67.2%)	44 (88%)	8 (16%)	–	–	–	31 (79.4%)	–	–		
Fatigue	11 (50%)	1 (5)	129 (54.2%)	12 (5.0%)	22 (44%)	2 (4%)	16 (45%)	4 (11%)	29 (72.5%)	8 (20.5%)	19 (61.3%)	4 (12.9%)	226 (54.5%)	31 (7.5%)
Nausea	13 (59%)	1 (5%)	130 (54.6%)	–	19 (38%)	1 (2%)	12 (35%)	0	28 (70%)	2 (5.1%)	17 (54.8)	0 (0%)	219 (52.8%)	4 (1.0%)
Anemia	9 (41%)	6 (27%)	147 (61.8%)	56 (23.5)	16 (32%)	5 (10%)	25 (72%)	16 (46%)	–	–	22 (70.9%)	5 (16.1%)	219 (52.8%)	88 (21.2%)
Alopecia	–	–	121 (50.8%)	–	–	–	–	–	–	–	–	–	121 (29.2%)	–
Diarrhea	5 (23%)	0	67 (28.2%)	–	13 (26%)	0	7 (20%)	0	25 (62.5%)	1 (2.6%)	10 (32.3%)	0 (0%)	127 (30.6%)	1 (0.2%)
Decreased neutrophil count	4 (18%)	3 (14%)	99 (41.6%)	64 (26.9)	–	–	12 (34%)	4 (11%)	–	–	5 (16.1%)	5 (16.1)	120 (28.9%)	76 (18.3%)
Decreased platelet count	6 (27)	2 (9%)	71 (29.8%)	14 (5.9%)	–	–	25 (72%)	10 (29%)	–	–	9 (29.1%)	2 (6.5%)	111 (26.7%)	28 (6.7%)
Vomiting	8 (36%)	0	61 (25.6%)	–	8 (16%)	0			14 (35%)	1 (2.6%)	6 (19.4%)	0 (0%)	97 (23.4%)	1 (0.2%)
Anorexia	5 (23%)	0	55	–	12 (24%)	1 (2%)	5 (14%)	0	16 (40%)	6 (15%)	–	–	93 (22.4%)	7 (1.7%)
Constipation	5 (23%)	0	78 (32.8%)	–	–	–	–	–	–	–	–	–	83 (20.0%)	0
Cough	7 (32%)	0	–	–	–	–	–	–	–	–	–	–	7 (1.7%)	0
Palpitations	7 (32%)	0	–	–	–	–	–	–	–	–	–	–	7 (1.7%)	0
Neuropathy peripheral	–	–	60 (25.2%)	–	2 (4%)	0	–	–	–	–	–	–	62 (14.9%)	0
Peripheral sensory neuropathy	–	–	60 (25.2%)	–	1 (2%)	0	–	–	–	–	–	–	61 (14.7%)	0
Arthralgia	–	–	58 (24.4%)	–	–	–	–	–	–	–	–	–	58 (14.0%)	0
Decreased white blood cell count	2 (9%)	1 (5%)	48	15 (6.3%)	2 (4%)	1	4 (12%)	0	–	–	–	–	56 (13.5%)	17 (4.1%)
Urinary tract infection	–	–	48 (20.2%)	–	–	–	–	–	–	–	–	–	48 (11.6%)	0
Hypertension	2 (9%)	1 (5)	–	–	2 (4%)	0	–	–	31 (77.5%)	13 (33.3%)	–	–	35 (8.4%)	14 (3.4%)
Abdominal pain	4 (18%)	0	–	–	3 (6%)	0	–	–	10 (33.3%)	1 (2.6%)	–	–	17 (4.1%)	1 (0.2%)
Dyspnea	13 (59%)	2 (9%)	–	–	–		–	–	–	–	–	–	13 (3.1%)	2 (0.4%)
Dizziness	9 (41%)	0	–	–	2	0	–	–	–	–	4 (12.9)	0	9 (2.2%)	0
Creatinine increased	7 (32%)	9 (41%)	–	–	–	–	–	–	–	–	–	–	7 (1.7%)	9
Myalgia	5 (23%)	1 (5%)	–	–	–	–	–	–	–	–	–	–	5 (1.2%)	1 (0.2%)
Bloating	5 (23%)	0	–	–	–	–	–	–	–	–	–	–	5 (1.2%)	0

Only AEs occurring in > 25% of patients are included in the table.

## Discussion

This is an up-to-date meta-analysis on the efficacy and safety of PARP inhibitors for patients with advanced or recurrent EC. We included 12 studies with a total of 1,594 patients, and the most reliable comparative information comes from the HRs of randomized trials. The main findings from the pooled analyses were as follows: (1) the results showed that the combination of PARP and PD-L1/PD-1 inhibitors could significantly prolong PFS versus placebo; (2) in the intention-to-treat population, there was no PFS/OS difference between PARP inhibitor monotherapy and placebo; (3) the ORR was notably elevated in the combined therapy group compared with PARP inhibitor monotherapy; and (4) the most common AEs included fatigue (226, 54.5%), nausea (219, 52.8%), anemia (219, 52.8%), diarrhea (127, 30.6%), and alopecia (121, 29.2%).

Recently, novel immunotherapeutic strategies have been proposed to enhance clinical efficiency. PARP inhibitors are among the most promising agents and have already demonstrated compelling results in ovarian cancer (28). Furthermore, as it has been reported that the accumulation of DNA damage caused by PARP inhibitors may reverse the immune escape by upregulating PD-L1, combining PD-1/PD-L1 inhibitors with PARP inhibitors is an attractive approach for EC patients (28). Our study suggests that the combination of PARP with PD-L1/PD-1 inhibitors could significantly prolong PFS versus placebo for advanced or recurrent EC patients, and ORR was notably elevated in the combined therapy group compared with PARP inhibitor monotherapy. Studies investigating PD-1/PD-L1 inhibitor monotherapy in MMRd EC revealed a median PFS ranging from 4.4 to 25.7 months, along with an ORR of 26.7% to 57.1% (5, 29, 30,), which were better than those reported in this meta-analysis. The outcomes of our study appear better than those of studies involving PD-1/PD-L1 inhibitors in pMMR EC, which indicated a median PFS of 1.8 to 1.9 months and an ORR of 3.0% to 13.4% (4, 29, 30). Several previous studies have found that the combination of PARP inhibitors, such as olaparib and niraparib, with PD-1/PD-L1 inhibitors might have modest clinical effects (17–19). The addition of the anti-PD-L1 antibody durvalumab with olaparib to platinum-based chemotherapy significantly improved PFS for patients with advanced or recurrent EC compared to durvalumab or chemotherapy alone, demonstrating a potential role for PARP inhibitors (18).

The efficacy of PARP inhibitors was also tested in association with other drugs, such as antiangiogenesis and AKT inhibitors (16, 21, 22). Some studies demonstrate that chronic hypoxia induced by antiangiogenic agents, such as bevacizumab, can lead to HRD within tumors, potentially enhancing sensitivity to PARP inhibitors (16, 31, 32). The combination of antiangiogenic agents with PARP inhibitors has also been assessed in a phase II trial (NCT03660826) (20). In this three-arm randomized trial, PFS was 3.8 months for cediranib alone, 2 months for olaparib, and 5.5 months for olaparib and cediranib. However, the between-arm differences were not statistically significant (20). In this meta-analysis, we included two trials that investigated the combination of antiangiogenic agents with PARP inhibitors. The pooled ORR was found to be 0.138 (95% CI = 0.072–0.203) for advanced or recurrent EC, which is similar to that observed with the combined therapy including a PD-1/PD-L1 inhibitor.

While generally manageable with successful management strategies, AEs occurred more frequently and remain a significant concern with combination immunotherapies. The results of this study indicated that the most commonly reported AEs of any grade included fatigue (226, 54.5%), nausea (219, 52.8%), anemia (219, 52.8%), diarrhea (127, 30.6%), and alopecia (121, 29.2%). Although the frequency of adverse events is typically higher in combination chemotherapies, grade ≥ 3 AEs were reported with low incidence, with the most frequent being anemia (88, 21.2%), decreased neutrophil count (76, 18.3%), fatigue (31, 7.5%), decreased platelet count (28, 6.7%), and decreased white blood cell count (17, 4.1%).

In the phase III DUO-E trial, the overall incidence of treatment-emergent AEs of grade ≥ 3 in the control, durvalumab, and durvalumab + olaparib arms was 56.4%, 54.9%, and 67.2%, respectively (18). Although there was a higher rate of AEs of grade ≥ 3 in the durvalumab + olaparib arm, the safety profiles were generally consistent with the known profiles of the individual components of the regimen (18). However, in the phase II DOMEC trial (19), the combination of durvalumab and olaparib was well tolerated, with no new safety profiles observed, consistent with those in previous studies (19, 29, 30).

Targeted therapies, such as olaparib, which inhibit PARP, induce synthetic lethality in tumor cells harboring HRD. The percentage of BRCA-mutated endometrial cancers is very low; therefore, HRD is an emerging concept in EC and appears to play a role in its pathogenesis. HRD is largely restricted to TP53-mutated ECs, and approximately 20%–40% of p53-abnormal tumors harbor HRD. The UTOLA trial suggests that olaparib may provide the greatest PFS benefit in the population with TP53 mutations (25).

For patients with pMMR disease, the magnitude of PFS benefit is lower, and the efficacy of PD-1 and PD-L1 inhibitors is variable. In the recurrent setting, the combination of pembrolizumab and lenvatinib is a promising therapeutic option for the pMMR/microsatellite-stable (MSS) subset, despite the significant morbidity associated with this pharmacological regimen. Moreover, exploratory subgroup analyses of the DUO-E trial suggest that the addition of maintenance olaparib to the combination of durvalumab plus chemotherapy may improve outcomes in the pMMR and PD-L1–positive patient populations (25). Consequently, assessing PD-L1 expression in recurrent pMMR/MSS cases could help identify patients who might benefit from the combination of PARP with ICIs (33).

There has been strong interest in utilizing a PARP inhibitor alone or in combination with other agents for managing EC. Several ongoing trials are testing the safety and effectiveness of PARP inhibitors in combination with chemotherapy (e.g., NCT04159155, NCT03603184), ICI (e.g., NCT04269200, NCT03016338, NCT03981796), and antiangiogenics (e.g., NCT03660826). Moreover, further biomarker-driven trials aimed at guiding the therapeutic selection and testing targeted agents are warranted.

This research has several limitations. First, owing to restricted data availability, subgroup analyses based on factors such as MMR status, PD-L1 expression, BRCA mutation, and HRD status were not feasible. Second, several clinical trials have not yet reached their final OS endpoints; thus, our OS analyses are based on a limited number of immature datasets and should be interpreted with caution. Updated analyses will be needed once these trials mature. Third, the majority of included trials were nonrandomized, single-arm, and unblinded, and therefore carried high risks of selection bias and performance bias. Further large-scale RCTs are required to confirm these results.

## Conclusion

In conclusion, our study provided preliminary evidence that the combination of PARP inhibitors with PD-L1/PD-1 inhibitors exhibits modest activity accompanied by substantial toxicity, particularly in heavily pretreated and predominantly pMMR advanced or recurrent EC. Definitive conclusions regarding survival benefits, however, require further phase III data and biomarker-driven analyses.

## Data Availability

The original contributions presented in the study are included in the article/**supplementary material**. Further inquiries can be directed to the corresponding author.

## Supplementary material

Supplementary Figure 1 Forest plot of PFS for PARP inhibitors combination therapy in advanced or recurrent EC (excluding DUO-E trial and RUBY trial). Forest plot of **(A)** overall population; **(B)** patients received the combined therapy with PD-1/PD-L1 inhibitor.

Supplementary Figure 2 Funnel plots for evaluating publication bias methods. **(A)** PFS (overall population); **(B)** PFS (PARP inhibitors combined with PD-1/PD-L1 inhibitor); **(C)** PFS (PARP inhibitors combined with PD-1/PD-L1 inhibitor, excluding DUO-E trial and RUBY trial); **(D)** PFS (overall population, excluding DUO-E trial and RUBY trial); **(E)** ORR (overall population).

Supplementary Figure 3 Pooled proportion of AEs incidence for PARP inhibitors combination therapy in EC. **(A)** overall incidence of AEs; **(B)** incidence of AEs of grade ≥3.
