# Supplementary figures and images for: Efficacy and safety of PARP inhibitors in advanced or recurrent endometrial cancer: a systematic review and meta-analysis
Source: Front Immunol. 2026 Jan 7;16:1659650. doi: 10.3389/fimmu.2025.1659650 (PMC12819624; doi:10.3389/fimmu.2025.1659650)

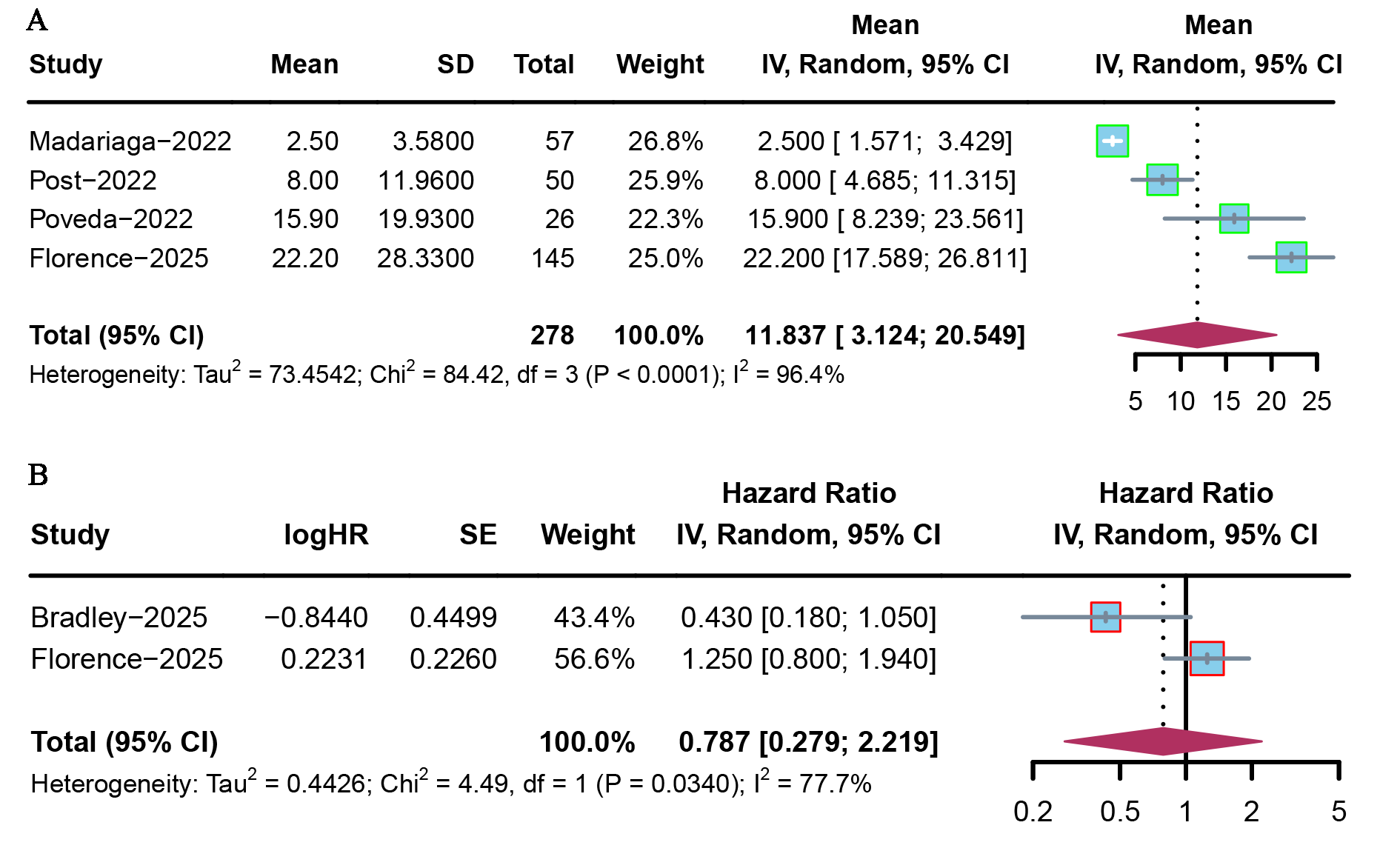

Supplement: Supplementary Figure 1 — Forest plot of PFS for PARP inhibitors combination therapy in advanced or recurrent EC (excluding DUO-E trial and RUBY trial). Forest plot of (A) overall population; (B) patients received the combined therapy with PD-1/PD-L1 inhibitor. [file Image1.tif]

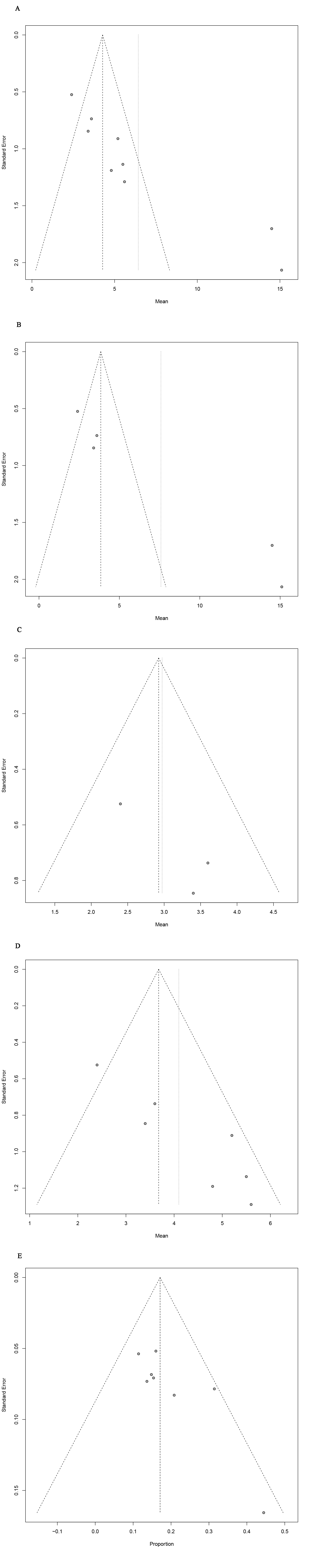

Supplement: Supplementary Figure 2 — Funnel plots for evaluating publication bias methods. (A) PFS (overall population); (B) PFS (PARP inhibitors combined with PD-1/PD-L1 inhibitor); (C) PFS (PARP inhibitors combined with PD-1/PD-L1 inhibitor, excluding DUO-E trial and RUBY trial); (D) PFS (overall population, excluding DUO-E trial and RUBY trial); (E) ORR (overall population). [file Image2.tif]

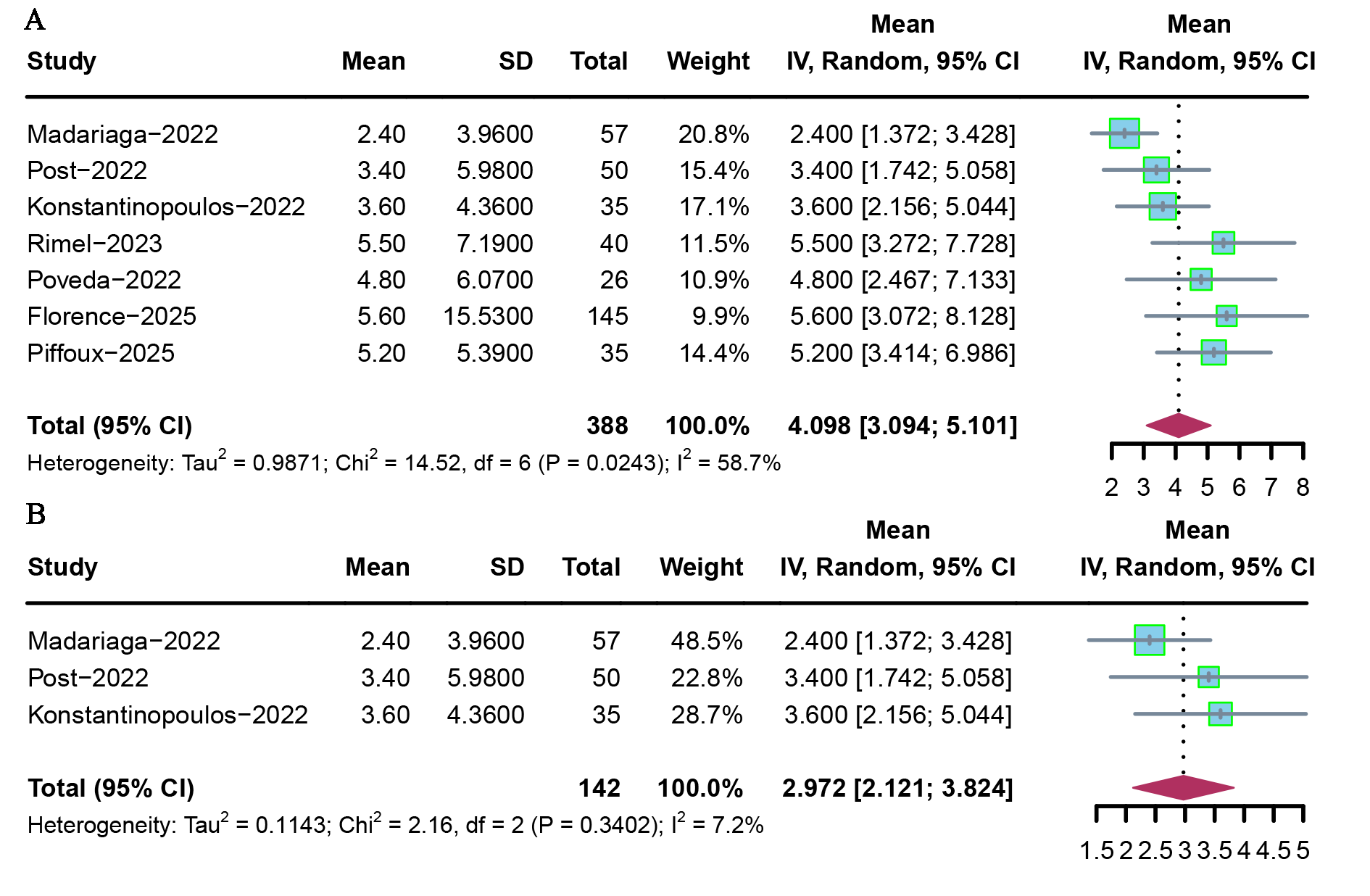

Supplement: Supplementary Figure 3 — Pooled proportion of AEs incidence for PARP inhibitors combination therapy in EC. (A) overall incidence of AEs; (B) incidence of AEs of grade ≥3. [file Image3.tif]
